# Diagnosis and treatment delay among pulmonary tuberculosis patients identified using the Taiwan reporting enquiry system, 2002–2006

**DOI:** 10.1186/1471-2458-9-55

**Published:** 2009-02-12

**Authors:** Hui-Ping Lin, Chung-Yeh Deng, Pesus Chou

**Affiliations:** 1 Community Medicine Research Center and Institute of Public Health, National Yang-Ming University, Taipei, Taiwan, Republic of China; 2 Nantou County Government Health Bureau, Nantou, Taiwan, Republic of China; 3 Institute of Hospital and Health Care Administration, National Yang-Ming University, Taipei, Taiwan, Republic of China

## Abstract

**Background:**

The tuberculosis reporting enquiry system was launched in Taiwan in 2001. Tuberculosis has been categorized as the third most important notifiable disease in Taiwan and the time required for reporting has been shortened to 7 days.

**Methods:**

A total of 114,827 cases were reported using the Taiwan enquiry system between 2002 and 2006; of these, 26,027 (22.7%) were finally diagnosed as not being tuberculosis, 7,005 (8.2%) were diagnosed as extra-pulmonary tuberculosis and 3,677 (3.2%) were not a first-time diagnosis of tuberculosis, and these cases were hence excluded. Diagnosis time was defined as the length of time between the first medical examination (including chest radiography, sputum smear or sputum culture) to the diagnosis of PTB; treatment time was defined as the period from the diagnosis of PTB to the initiation of treatment. Using the cut-off at the 75^th ^percentile, a period of longer than 9 days was defined as a *diagnosis delay *and a period of longer than 2 days as a *treatment delay*. Multiple logistic regression analysis was applied to analyze the risk factors associated with these delays.

**Results:**

During the five-year study period, among the 78,118 new PTB patients reported in Taiwan, the mean diagnosis and treatment times were 12 and 5 days and the median times 1 day and 0 days, respectively. In total, 24.9% of the new PTB patients' diagnosis time delays were longer than 9 days and 20.3% of the patients' treatment time delays were longer than 2 days. The main factors associated with diagnosis delay included age, reporting year, living with family and a positive sputum culture (*p *< 0.0001); the risk factors significantly associated with treatment delay were increased age, an aboriginal ethnic background, a positive sputum culture and diagnosis at a non-medical center (*p *< 0.0001).

**Conclusion:**

The Taiwan TB reporting enquiry system has successfully increased the confirmed PTB reporting rate from 64.4% to 71.5%. Greater age and a positive sputum culture were both found to significantly increase both diagnosis and treatment delays; treatment delay is also significantly affected by the patient having an aboriginal ethnic background and being diagnosed at a non-medical center.

## Background

Diagnosis and treatment delay of tuberculosis (TB) may result in more extensive disease and more complications, which lead to higher mortality and an increased period of infectivity in the community [1-3]. In order to control and manage TB cases more efficiently, simplify bureaucratic procedure and prevent duplication of tasks of first-line healthcare workers, the national TB database was established in Taiwan in 1994, and was transferred to a computer-based system in 1996; owing to technological advances, increased internet access and data control, it has been widely used since 2002. In accordance with the Taiwanese Communicable Disease Control Act, all suspected/confirmed TB cases must be reported to the CDC after verification by a physician. To enable this, the TB reporting enquiry system was launched in Taiwan in 2001 [4] and has since been strengthened to improve the efficiency of reporting via the websites of medical care institutions.

The National Health Insurance (NHI) program was established in Taiwan, in 1995, and the proportion of the population insured reached 98.29% in 2006 [5]. TB patients can receive medical examinations and anti-TB medication in any public center, private clinic or hospital under the NHI program [6]. Qualified doctors report cases via the reporting enquiry system based on laboratory tests, chest radiography, and clinical symptoms, e.g., persistent coughs.

The Communicable Disease Control Act was amended and promulgated on January 20, 2004, and now consists of 75 articles in seven chapters. TB was categorized as the third most important notifiable disease in Taiwan, and the time required for reporting was shortened to 7 days [5].

## Methods

### Setting and study population

The Centers for Disease Control, Taiwan, Republic of China, gave permission for this research to be performed. A total of 114,827 cases of TB were reported to the Taiwan enquiry system between January 1, 2002 and December 31, 2006; the yearly totals were 24,771, 22,337, 24,135, 22,718 and 20,784 in 2002–2006, respectively, which gives notification rates of 109.99, 98.81, 106.37, 99.77 and 90.85 per 100,000 people, respectively. Of these cases, 26,027 (22.7%) were finally diagnosed as not having TB, 7,005 (8.2%) were diagnosed with extra-pulmonary TB and 3,677 (3.2%) were not a first-time diagnosis of TB, and hence these cases were excluded, leaving a final total of 78,118 new pulmonary TB cases (PTB) that were eventually included in this study. The total number of new PTB cases was therefore 15,954, 14,875, 16,463, 15,916 and 14,851, 2002–2006, respectively. Thus, the confirmed PTB rates were 64.4%, 66.6%, 68.2%, 70.1% and 71.5%, or, in other words, the confirmed PTB notification rates were 70.84, 65.80, 72.56, 69.90 and 64.92 per 100,000 people, 2002–2006, respectively.

### Definitions

The criteria for the diagnosis of PTB patients were a positive sputum smear or culture, an aberrant chest radiograph or clinical symptoms such as a persistent cough; the criteria for the diagnosis of "not TB" were change diagnosis into not PTB, for example pneumonia or lung cancer. Diagnosis time was defined as the length of time between the first medical examination (including chest radiography, sputum smear or sputum culture) and the diagnosis of PTB; treatment time was defined as the period from the diagnosis of PTB to the initiation of treatment. Using the cut-off at the 75^th ^percentile, a period of longer than 9 days was defined as a *diagnosis delay *and a period of longer than 2 days as a *treatment delay*.

### Statistical analysis

SPSS version 13.0 was used for data analysis in order to calculate how the TB reporting enquiry system is affected by variables such as sex, age, ethnicity, reporting year, site of disease, living conditions, sputum smear status, sputum culture status, chest radiography and the hospital level at which the diagnosis took place. Bivariate analyses were performed together with a computation of descriptive statistics; two-sample t-tests and one-way ANOVA were used to compare the mean times, and univariate and multiple logistic regressions were used to assess the potential risk factors of diagnosis delay and treatment delay. A *p *value of less than 0.05 was considered to indicate statistical significance.

## Results

Table 1 shows the characteristics of the pulmonary tuberculosis patients diagnosed in Taiwan, 2002–2006. During the five-year period of study, 78,118 new PTB patients were reported; of these, 54,187 (69.4%) were male. The mean age of the patients was 63.3 years, ranging from 1–108 years; in total, 42,648 (54.6%) patients were aged over 65 at the time of diagnosis. Among the study subjects, 3.4% of patients were from abroad, 3.2% were of an aboriginal ethnic background, 16.9% lived alone, 34.8% had a positive sputum smear, 40.8% had a positive sputum culture, 87.2% had an aberrant chest radiograph, 18.8% had positive sputum smear and sputum culture results, 31.5% had both a positive sputum smear and an aberrant chest radiograph, 38.7% had both a positive sputum culture and an aberrant chest radiograph, 17.5% had positive sputum smear and sputum culture results and an aberrant chest radiograph, and 36.0% were diagnosed at a medical center. Overall, 72.2% of the patients completed their treatment. In total, 23.3% patients showed that they had died within a certain period of time after diagnosis/treatment, but of these, only 3.0% died of TB.

**Table 1:** Characteristics of pulmonary tuberculosis patients, Taiwan, 2002 – 2006.

		Diagnosis Time (days)		Treatment Time (days)	
			
	N (%)	Mean ± SD	*p *value	Mean ± SD	*p *value
**Total**	**78,118**	**12 **± **28**		**5 ± 29**	
Sex			0.05		0.001
Male	54,187 (69.4)	12 ± 28		5 ± 30	
Female	23,931 (30.6)	11 ± 27		4 ± 26	
Age at diagnosis, mean age (range) 63.3 (1 – 108)			< 0.001		< 0.001
< 35	10,386 (13.3)	7 ± 22		4 ± 24	
35 – 65	25,084 (32.1)	9 ± 26		5 ± 34	
> 65	42,648 (54.6)	14 ± 29		5 ± 26	
Reporting year			< 0.001		< 0.001
2002	15,954 (20.4)	8 ± 27		7 ± 40	
2003	14,875 (19.1)	8 ± 25		5 ± 32	
2004	16,463 (21.1)	12 ± 28		4 ± 25	
2005	15,916 (20.4)	14 ± 28		4 ± 24	
2006	14,851 (19.0)	16 ± 30		4 ± 23	
Ethnicity			< 0.001		< 0.001
Taiwanese	72,957 (93.4)	12 ± 28		5 ± 29	
Aboriginal	2,530 (3.2)	9 ± 23		9 ± 38	
Abroad	2,631 (3.4)	6 ± 23		4 ± 21	
Living condition			< 0.001		< 0.001
Alone	13,202 (16.9)	10 ± 26		6 ± 36	
With family	50,096 (64.1)	11 ± 27		5 ± 28	
Unknown	14,820 (19.0)	16 ± 30		4 ± 25	
Sputum smear status			< 0.001		< 0.001
Negative	43,170 (55.3)	16 ± 31		5 ± 27	
Positive	27,209 (34.8)	5 ± 18		4 ± 29	
Unknown	7,739 (9.9)	10 ± 30		6 ± 35	
Sputum culture status			< 0.001		< 0.001
Negative	20,535 (26.3)	8 ± 26		4 ± 26	
Positive	34,721 (40.8)	20 ± 32		5 ± 27	
Unknown	27,582 (32.4)	3 ± 17		6 ± 34	
Chest radiograph			< 0.001		< 0.001
Normal	1,905 (2.4)	21 ± 30		6 ± 32	
Aberrant, no cavity	51,320 (65.7)	13 ± 29		5 ± 28	
Aberrant, cavity	16,770 (21.5)	6 ± 21		4 ± 28	
Unknown	8,123 (10.4)	14 ± 28		7 ± 36	
Diagnosed at a medical center			< 0.001		0.601
Yes	28,151 (36.0)	13 ± 30		5 ± 30	
No	49,967 (64.0)	11 ± 26		5 ± 28	

SD = standard deviation

The mean diagnosis and treatment times were 12 and 5 days, respectively, and the median times were 1 day and 0 days, respectively. The mean diagnosis and treatment times were longer for male patients than for female patients (*p *≤ 0.05), and diagnosis and treatment times increased with age (*p *< 0.001). Diagnosis time also increased with reporting year (*p *< 0.001), but treatment time decreased with reporting year (*p *< 0.001). Patients from abroad had the shortest mean diagnosis and treatment times in comparison with those from Taiwan and those of an aboriginal ethnic background (*p *< 0.001), and those who of aboriginal ethnic background had the longest mean treatment time (*p *< 0.001). Those who lived alone had the shortest mean diagnosis time (*p *< 0.001) but the longest mean treatment time (*p *< 0.001). Patients whose sputum smears were positive had the shortest mean diagnosis and treatment times (*p *< 0.001), whereas those whose sputum cultures were positive had the longest mean diagnosis and treatment times (*p *< 0.001). Patients with an aberrant chest radiograph had the shortest mean diagnosis and treatment times (*p *< 0.001). Patients diagnosed at a medical center had a longer mean diagnosis time as compared with those diagnosed at a non-medical center (*p *< 0.001).

Table 2 shows the multiple logistic regression analysis of the factors associated with diagnosis delay and treatment delay among PTB patients in Taiwan, 2002–2006. In total, 19,413 (24.9%) new PTB patients experienced a diagnosis delay and 14,270 (20.3%) patients experienced a treatment delay, and opposite trends in diagnosis delay and treatment delay were seen between 2002 and 2006. Those of an aboriginal ethnic background experienced greater treatment delay but less diagnosis delay. As a whole, the patterns of the rates for diagnosis delay and treatment delay for the associated variables were similar to the patterns of the mean diagnosis time and treatment time, as shown in Table 1.

**Table 2:** Multiple logistic regression analysis of the factors associated with delay among PTB patients, Taiwan, 2002 – 2006.

	Diagnosis Delay (time > 9 days)					Treatment Delay (time > 2 days)				
		
Variables	N	Rate (%)	*p *value	AOR	95% CI	N	Rate (%)	*p *value	AOR	95% CI
**No. of patients**	**19,413**	**24.9**				**14,270**	**20.3**			
Sex			0.006					0.002		
Male	13,619	25.1		1		10,066	20.6		1	
Female	5,794	24.2		1.04	0.99 – 1.08	4,204	19.6		0.93	0.90 – 0.97
Age at diagnosis, years			< 0.001					< 0.001		
< 35	1,386	13.3		1		1,727	18.5		1	
35 – 65	4,875	19.4		1.77	1.64 – 1.90	4,609	19.9		1.12	1.05 – 1.19
> 65	13,152	30.8		2.99	2.79 – 3.21	7,934	21.0		1.16	1.09 – 1.23
Reporting year			< 0.001					< 0.001		
2002	2,489	15.6		1		3,311	25.7		1	
2003	2,669	17.9		1.04	0.97 – 1.11	2,575	20.4		0.72	0.68 – 0.76
2004	4,281	26.0		1.42	1.33 – 1.51	2,849	18.5		0.64	0.60 – 0.68
2005	4,929	31.0		1.59	1.49 – 1.70	2,877	19.1		0.66	0.62 – 0.71
2006	5,041	33.9		2.11	1.65 – 2.71	2,658	18.7		0.64	0.50 – 0.81
Ethnicity			< 0.001					< 0.001		
Taiwanese	18,594	25.5		1		13,176	19.9		1	
Aboriginal	528	20.9		1	0.90 – 1.12	746	31.4		1.92	1.76 – 2.11
Abroad	291	11.1		0.81	0.70 – 0.93	348	20.4		1.13	1.00 – 1.29
Living condition			< 0.001					< 0.001		
Alone	2,834	21.5		1		2,460	22.3		1	
With family	11,594	23.1		1.11	1.05 – 1.17	9,161	20.3		0.90	0.85 – 0.95
Unknown	4,985	33.6		0.90	0.71 – 1.16	2,649	18.7		0.93	0.73 – 1.19
Sputum smear status			< 0.001					< 0.001		
Negative	15,121	35.0		1		8,321	21.3		1	
Positive	2,793	10.3		0.15	0.14 – 0.16	4,555	18.0		0.78	0.75 – 0.82
Unknown	1,499	19.4		0.61	0.57 – 0.66	1,394	23.8		0.99	0.92 – 1.05
Sputum culture status			< 0.001					< 0.001		
Negative	3,643	17.7		1		3,316	17.3		1	
Positive	14,048	40.5		4.71	4.49 – 4.93	6,913	21.6		1.44	1.38 – 1.51
Unknown	1,722	7.5		0.56	0.53 – 0.60	4,041	21.3		1.22	1.16 – 1.29
Chest radiograph			< 0.001					< 0.001		
Normal	860	45.1		1		393	22.9		1	
Aberrant, no cavity	14,070	27.4		0.56	0.50 – 0.63	9,592	20.7		0.90	0.80 – 1.01
Aberrant, cavity	2,034	12.1		0.26	0.23 – 0.29	2,590	16.7		0.68	0.60 – 0.77
Unknown	2,449	30.1		0.66	0.58 – 0.74	1,695	25.4		1.06	0.94 – 1.21
Diagnosed at a medical center			< 0.001					0.003		
Yes	8,388	29.8		1		4,966	19.7		1	
No	11,025	22.1		0.69	0.67 – 0.72	9,304	20.7		1.09	1.04 – 1.13

PTB = pulmonary tuberculosis; AOR = adjusted odds ratio; CI = confidence interval.

After controlling for the covariates, the significant risk factors for diagnosis delay were increasing age, increasing reporting year, living with family (as compared with living alone), a negative sputum smear, a positive sputum culture, a normal chest radiograph and being diagnosed at a medical center. The adjusted odds ratios (AORs) and their 95% confidence intervals (95% CIs) are shown in Table 2.

After controlling for the covariates, the significant risk factors for treatment delay were being male, of greater age, decreasing reporting year, an aboriginal ethnic background or from abroad, living alone, a negative sputum smear, a positive sputum culture, a normal chest radiograph, and being diagnosed at a non-medical center. The AORs and their 95% CIs are shown in Table 2.

## Discussion

The majority of studies in this area have involved the interviewing of patients via questionnaires and have collected patients' self-reported periods of time between the onset of TB symptoms and their first visit to a medical provider (patient delay) and between their first contact with a qualified doctor and the initiation of TB treatment (health system delay). This study is the first to analyze data obtained from a nationwide database, the TB reporting enquiry system. One limitation of this study is that data from which to estimate the time between the onset of coughing and the first medical examination are not available, and only limited data are available for analysis. The completeness of data reporting was not perfect in the early period of use of the reporting enquiry system, and missing data (unknown data) was therefore inevitable.

Adjusting for other variables, being female, in comparison with being male, had a protective factor with respect to treatment delay. This finding is similar to those of other studies conducted in Kampala [7] and southern India [8]; however, some other studies have identified being female as having adverse consequences in terms of a longer delay in diagnosis [6,9] and in treatment [10-12].

Similar to the findings of many other studies, diagnosis and treatment delays were found to significantly increase with age [7,9,13-17]. In Taiwan, the life expectancy in 2006 for men and women was 75.1 and 81.9 years, respectively [5]. In this study, the mean age was 63.3 years, and 54.6% of patients were aged over 65 years. Many studies [13,18-20] have stratified and only studied those who were aged over 65, but because the sample size of this study was large enough, we chose to divide the rest of the patients into two groups, those aged under 35 and those aged between 35 and 65. A dose-response effect of age was found in this study, and the older the patient, the more serious the consequences of diagnosis and treatment delays.

Being of an aboriginal ethnic background was found to be a significant risk factor associated with treatment delay as compared with the general Taiwanese population (1.92 times higher). Taiwan has impelled the aboriginal community to attend health screening since 2001: aboriginal people aged 15 years and over are urged to take advantage of the free chest radiograph inspections offered every year, and diagnosis would then not be a problem. However, the medical service resources in aboriginal areas are relatively deficient, leading to delays in treatment time. It should be noted that the mortality rate and incidence of TB are much greater in aboriginal communities than in non-aboriginal areas in Taiwan [19,21-24], and this may have an influence on prevention and treatment [25]. In contrast, being from abroad was found to be a significant protective factor for diagnosis delay. Some studies have also shown that those from abroad have a shorter health care system delay [18,26].

A total of 27,209 PTB patients had a positive sputum smear, and of these, 19.7% of sputum cultures were negative. One possible reason for this may be the density of the sputum collected. A positive sputum culture was found to be associated with increased delays only when the sputum smear was negative, not when it was positive. Some studies have found that a negative sputum smear is associated with greater diagnosis and treatment delays [14,20,27-29], and one study found that a negative sputum smear was negatively associated with diagnosis delay [15].

In this study, one possible reason for the opposite trends of diagnosis and treatment delay from 2002 to 2006 may be that the method of TB diagnosis was chest radiography prior to 2002, but gradually moved towards laboratory testing from 2002 onwards. The process of testing may postpone the time of diagnosis, but on the other hand, Taiwan CDC and the NHI cooperate with the policy that "the TB patient who is not notified does not pay," which prevents the hindering of treatment by doctors.

Living with family as compared with living alone resulted in a significantly shorter treatment delay but a 1.11 times higher diagnosis delay. Two previous studies found that being single was a positively-associated risk factor of diagnosis delay, namely studies performed in Ethiopia [30] and Botswana [16]. Diagnosis at a non-medical center was found to result in a significantly shorter diagnosis delay but a 1.09 times higher treatment delay. Some previous studies have found that an initial visit to a hospital was a negatively-associated risk factor of diagnosis delay [7,17,31,32]; furthermore, it has also been found that an initial visit to a governmental low-level healthcare facility, a traditional/unqualified practitioner or a private practitioner is a positively-associated risk factor of diagnosis delay [6,16,33,34].

## Conclusion

The Taiwan TB reporting enquiry system has successfully increased the confirmed PTB reporting rate from 64.4% to 71.5%. Both greater age and a positive sputum culture significantly increase both diagnosis and treatment delays; treatment delay is also significantly affected by the patient having an aboriginal ethnic background and by diagnosis at a non-medical center.

## Competing interests

The authors declare that they have no competing interests.

## Authors' contributions

HPL acquired and analyzed the data and wrote the manuscript; CYD joined in with discussion of the study design and data analysis; PC was in charge of the study design and supervised the whole research project. All three authors approved this manuscript.

## Pre-publication history

The pre-publication history for this paper can be accessed here:


